# Global Comparison of COVID-19 Vaccination Rates among Psoriasis Patients

**DOI:** 10.3390/life14101297

**Published:** 2024-10-13

**Authors:** Edwin Korouri, Charlotte Jeong, Hannah Peterson, Fernando Valenzuela, Ricardo Romiti, Johannes A. Didaskalu, Alexander Egeberg, Hazel H. Oon, Lara Valeska Maul, Paige Kingston, Kathryn Lee, Margaret Y. Huang, Danielle Yee, Kevin Artiga, Rosario Aguero, Julia-Tatjana Maul, April W. Armstrong

**Affiliations:** 1 Chicago Medical School, Rosalind Franklin University of Medicine and Science, North Chicago, IL 60064, USA; 2 College of Medicine, University of Arkansas for Medical Sciences, Little Rock, AR 72205, USA; 3 Division of Dermatology, Department of Medicine, University of California, Los Angeles, CA 90095, USA; 4 Department of Dermatology, University of Chile, Santiago 8330111, Chile; 5 Centro Internacional de Estudios Clínicos, Probity Medical Research, Santiago 8420383, Chile; 6 Department of Dermatology, School of Medicine, University of São Paulo, São Paulo 05508-220, Brazil; 7 Faculty of Medicine, University of Zurich, 8006 Zurich, Switzerland; 8 Department of Dermatology, Bispebjerg Hospital, 2400 Copenhagen, Denmark; 9 Department of Clinical Medicine, University of Copenhagen, 1172 Copenhagen, Denmark; 10 National Skin Centre, Singapore 308205, Singapore; 11 Skin Research Institute of Singapore (SRIS), Singapore 308232, Singapore; 12 Department of Dermatology, University Hospital Zurich, 8091 Zurich, Switzerland; 13 Department of Population and Public Health Sciences, Keck School of Medicine, University of Southern California, Los Angeles, CA 90007, USA

**Keywords:** psoriasis, biologics, vaccination, global, COVID-19

## Abstract

(1) Background: The purpose of this study is to compare the rate of COVID-19 vaccination among psoriasis patients internationally and to correlate it with their treatment regimens. (2) Methods: We conducted a cross-sectional study from January 2021 to October 2022 among adults in the United States (US), Chile, China, Switzerland, and Singapore using the Global Healthcare Study on Psoriasis survey. (3) Results: A total of 310 psoriasis patients in the US (98), Chile (32), China (80), Switzerland (39), and Singapore (61) were surveyed. Of these, 248 patients (80.0%) were vaccinated at least once for COVID-19 (Chile: 100%, Singapore: 100%, US: 93.9%, Switzerland: 69.2%, China: 45.0%). Compared with other countries, patients in China were 89% less likely to report at least one COVID-19 vaccination (1 − 0.11 = 0.89; OR 0.11; 95% CI: 0.03–0.48), and patients in Switzerland were 80% less likely (1 − 0.20 = 0.80; OR 0.20; 95% CI: 0.05–0.79). Compared with patients on biologics, patients on topicals were 10.9 (95% CI: 2.1–56.6) times more likely to report at least one COVID-19 vaccination, and patients on oral systemics were 7.2 times more likely (95% CI: 1.6–31.6). (4) Conclusions: Country of residence and treatment regimen are associated with different COVID-19 vaccination rates in psoriasis patients.

## 1. Introduction

Psoriasis is a chronic inflammatory disease that affects 2–3% of the global population [1]. Treatment for psoriasis is typically stratified by the severity of symptoms. Mild psoriasis may be treated with topical medications alone. However, moderate-to-severe psoriasis and involvement of the hands, feet, scalp, nails, or genitals may require phototherapy, biologics, or non-biologic systemics.

During the COVID-19 pandemic, vaccination against SARS-CoV-2 became an important tool to reduce COVID-19 infection, hospitalization, and death. Some patients on immunomodulatory biologics were uncertain of the safety of COVID-19 vaccines while on biologics. However, dermatologic societies in the United States (US) and European Union (EU) recommended that psoriasis patients on biologics receive non-live-attenuated vaccines. In the US, the National Psoriasis Foundation (NPF) recommended continuing the use of biologics and oral systemic medications for patients vaccinated with an mRNA vaccine [2,3,4,5]. In the EU, psoriasis patients were recommended to be fully vaccinated for COVID-19 with a booster while receiving any type of systemic therapy [4,5,6].

Even with evidence supporting the safety and efficacy of COVID-19 vaccines, some patients chose to avoid vaccination [7,8]. Although rates of COVID-19 vaccination among the general public have been reported, there is a lack of data comparing COVID-19 vaccination rates among psoriasis patients in different countries and comparing COVID-19 vaccination rates by treatment type for psoriasis patients globally. It is necessary for dermatologists worldwide to better understand the global rate of COVID-19 vaccination among psoriasis patients to elucidate opportunities that optimize the care for psoriasis patients and identify potential gaps in case of future pandemics.

The Global Healthcare Study on Psoriasis (GHSP) is an independent project collaborating with the Global Psoriasis Atlas (GPA), International Federation of Psoriasis Associations (IFPA), and Skin Inflammation and Psoriasis International Network (SPIN), among others, to identify and improve standards of psoriasis care worldwide. The GHSP aims to gather a growing cohort of global psoriasis patients that offers valuable insights into patient characteristics and comparisons with the general population. Our study aims to compare the rate of COVID-19 vaccination among (1) psoriasis patients internationally and (2) psoriasis patients on various treatment regimens internationally.

## 2. Materials and Methods

### 2.1. Data Source and Study Population

The GHSP is an international, longitudinal observational study that collects data on the standards of psoriasis care [9,10,11]. We utilized the GHSP to collect data from psoriasis patients regarding psoriasis treatments and the number of COVID-19 vaccinations received. Data were collected from 6 countries: Brazil, Chile, China, Singapore, Switzerland, and the US. Dermatologists and members of their medical team recruited psoriasis patients at outpatient, inpatient, and telehealth dermatology visits to participate in the GHSP. Patients responding to the GHSP reported demographic and clinical characteristics such as age, sex, country of residence, psoriasis treatment, and comorbidities. Beginning in December 2020, the GHSP collected data on the number of times a patient was vaccinated against COVID-19, prior COVID-19 diagnosis, and brand of vaccine received. We excluded psoriasis patients who were unable to receive COVID-19 vaccination due to pending government approval of the vaccine or inadequate vaccine supply at the time of data collection.

### 2.2. Data Analysis

This was a multicenter, multicountry analytical cross-sectional study conducted in Brazil, Chile, China, Singapore, Switzerland, and the US between 23 December 2020 and 3 October 2022. Brazil was excluded from the study because data were collected prior to vaccine approval and availability in the country.

Descriptive analysis of patient age, sex, race, education, insurance, employment, psoriasis severity, psoriasis treatment type, Charlson comorbidity index (CCI), previous COVID-19 diagnosis, and COVID-19 vaccination status was performed to describe the clinical and demographic characteristics of the cohort. Analysis of variance and chi-squared tests were used to calculate differences in characteristics among each of the five countries reporting survey responses to COVID-19-related questions.

A multivariable logistic regression was used to understand the association between COVID-19 vaccination and country of residence for adult psoriasis patients. Individual multivariable logistic regressions compared the vaccination rate of each country to the remainder of the countries together (e.g., US compared with Chile, China, Singapore, and Switzerland combined). All multivariable analyses were adjusted for age, sex, race, education, insurance, CCI, and previous COVID-19 diagnosis. The independent variable was defined as country of residence. The dependent variable was defined as at least one COVID-19 vaccination at the time of survey.

A second multivariable logistic regression was performed to evaluate the association between COVID-19 vaccination and psoriasis treatment type for adult psoriasis patients. Multivariable analyses were adjusted for country of residence, age, sex, race, education, insurance, CCI, and previous COVID-19 diagnosis. The independent variable was defined as the psoriasis treatment type (topicals, phototherapy, oral systemics, and biologics). The dependent variable was defined as at least one COVID-19 vaccination at the time of survey.

Statistical significance was determined at *p* < 0.05. All statistical analyses were performed using STATA 17.0 (StataCorp LP, College Station, TX, USA).

## 3. Results

### 3.1. Overall Cohort Characteristics

A total of 310 adult psoriasis patients across Chile, China, Switzerland, Singapore, and the US were analyzed regarding the number of times they had been vaccinated for COVID-19. The US reported 98 surveys (n = 98/310, 31.6%); China, 80 (n = 80/310, 25.8%); Singapore, 61 (n = 61/310, 19.7%); Switzerland, 39 (n = 39/310, 12.6%); and Chile, 32 (n = 32/310, 10.3%). Among all patients globally, 12.6% (n = 39/310) of patients were using topical therapy alone, 6.8% (n = 21/310) using phototherapy, 16.1% (n = 50/310) using oral systemics, and 56.8% (n = 176/310) using biologics. Demographic characteristics of the included patients are listed in Table 1.

**Figure 1:**
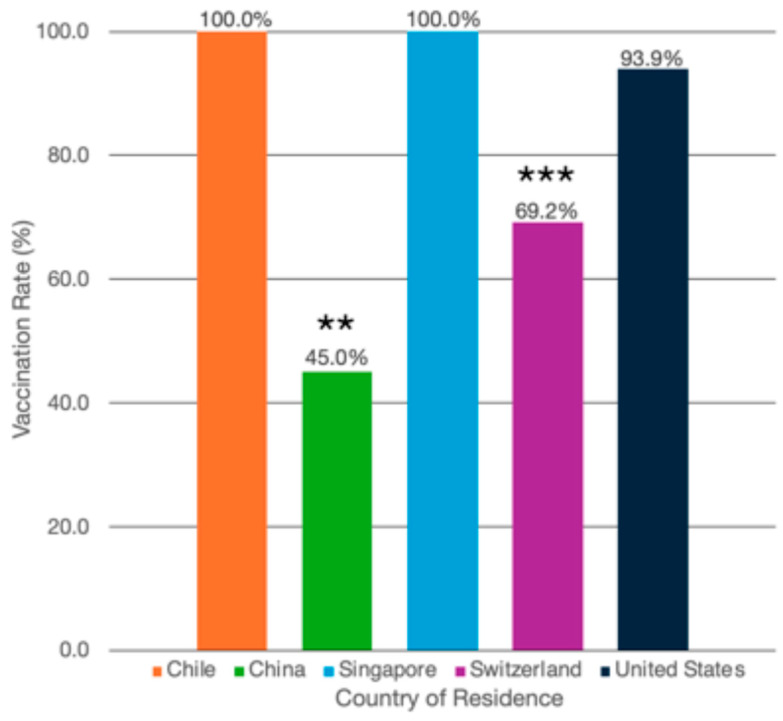
COVID-19 vaccination rates by country of residence. COVID-19 vaccination rate (as percentages) among psoriasis patients living in Chile (n = 32/32, 100.0%), China (n = 36/80, 45.0%), Singapore (n = 61/61, 100.0%), Switzerland (n = 27/39, 69.2%), and the United States (n = 92/98, 93.9%). ** Patients in China were significantly less likely to have had at least one vaccination compared with patients living in the rest of the countries combined (OR: 0.11, 95% CI: 0.03–0.48, *p* = 0.003). *** Patients in Switzerland were significantly less likely to have had at least one vaccination compared with patients living in the rest of the countries combined (OR: 0.20, 95% CI: 0.05–0.79, *p* = 0.022).

**Figure 2:**
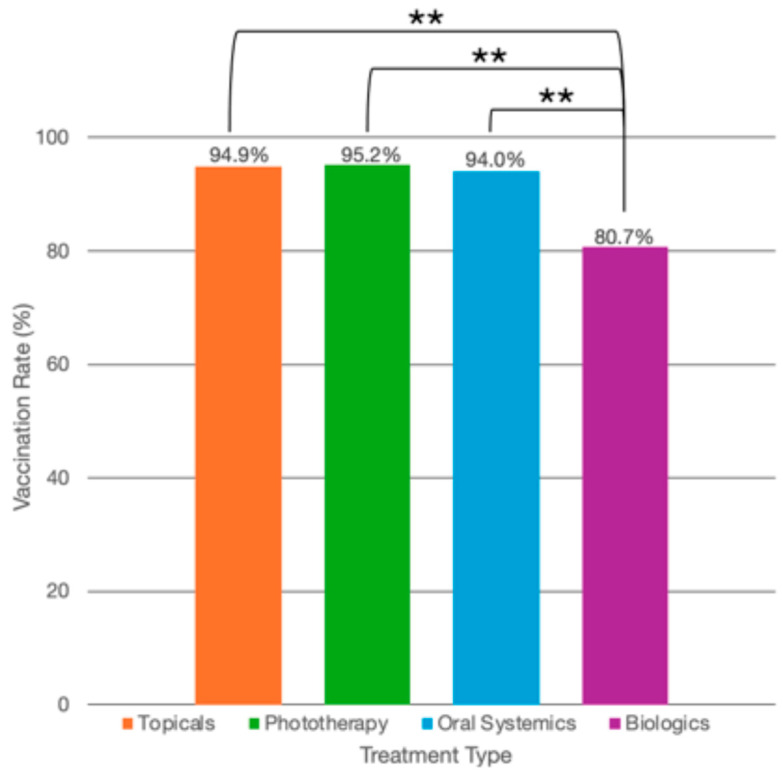
COVID-19 vaccination rates by treatment type. COVID-19 vaccination rates (as percentages) among psoriasis patients using topical therapy alone (n = 37/39, 94.9%), phototherapy (n = 21/21, 95.2%), oral systemics (n = 47/50, 94.0%), and biologics (n = 142/176, 80.7%). ** Patients on topical therapy alone were significantly more likely to have received at least one COVID-19 vaccination compared with those using biologics (OR: 7.2, 95% CI: 1.6–31.6, *p* = 0.009). Patients on oral systemics were significantly more likely to have received at least one COVID-19 vaccination than those on biologics (OR: 10.9, 95% CI: 2.1–56.6, *p* = 0.004). Patients using phototherapy had similar vaccination rates as those using biologics (OR: 2.4, 95% CI: 0.18–32.34, *p* = 0.506).

Among psoriasis patients globally, 80% of patients (n = 248/310) reported being vaccinated at least once for COVID-19. By country, Chile reported 100% (n = 32/32) of psoriasis patients as being vaccinated; China, 45.0% (n = 36/80); Singapore, 100% (n = 61/61); Switzerland, 69.2% (n = 27/39); and the US, 93.9% (n = 92/98) (Table 1 and Table 2, Figure 1). By psoriasis treatment type, 94.9% (n = 37/39) of patients on topical medications reported being vaccinated at least once for COVID-19; on phototherapy, 95.2% (n = 20/21); on oral systemics, 94.0% (n = 47/50); and on biologics, 80.7% (n = 142/176) (Table 3, Figure 2).

Of the patients who reported COVID-19 vaccination, 207 (83.5%) reported the brand they had been vaccinated with. The most commonly reported vaccination brands globally were Pfizer (63.7%), Moderna (20.8%), multiple brands (4.3%), Coronavac (3.9%), Covilo (3.9%), Johnson & Johnson (2.4%), AstraZeneca (0.5%), and Zhifei (0.5%).

By country, Chile reported Pfizer (80.7%), multiple brands (16.1%), and AstraZeneca (3.2%); China, Sinovac (47.1%), Sinopharm (47.1%), and Zifivax (5.8%); Singapore, Pfizer (78.7%), Moderna (19.7%), and multiple brands (1.6%); Switzerland, Pfizer (77.8%) and Moderna (22.2%); and the US, Pfizer (58.4%), Moderna (32.6%), Johnson & Johnson (5.6%), and multiple brands (3.4%).

### 3.2. Association between Country of Residence and COVID-19 Vaccination

Compared with Chile, Singapore, Switzerland, and the US, patients in China were 89% less likely to have received at least one COVID-19 vaccination (1 − 0.11 = 0.89; OR: 0.11, 95% CI: 0.03–0.48, *p* = 0.003). Patients in Switzerland were 80% less likely to have received at least one COVID-19 vaccination (1 − 0.20 = 0.80; OR: 0.20, 95% CI: 0.05–0.79, *p* = 0.022), with vaccination rates limited by data collection early in the pandemic. Compared with other countries, psoriasis patients in the US did not have a significant difference in COVID-19 vaccination rate (*p* = 0.248). Of note, multivariable logistic regressions comparing vaccination rates between Chile and the other countries were not included because all 32 patients who responded to the survey in Chile were vaccinated. As well, all 61 patients who responded to the survey in Singapore were vaccinated. Thus, an adjusted odds ratio could not be calculated for Chile and Singapore. The results of the multivariable analyses by country for COVID-19 vaccination are presented in Table 2A–E.

**Table 2:** (**A**). Multivariable logistic regression of the association between COVID-19 vaccination status and residence in Chile compared with other countries. (**B**) Multivariable logistic regression of the association between COVID-19 vaccination status and residence in China compared with other countries. (**C**) Multivariable logistic regression of the association between COVID-19 vaccination status and residence in Singapore compared with other countries. (**D**) Multivariable logistic regression of the association between COVID-19 vaccination status and residence in Switzerland compared with other countries. (**E**) Multivariable logistic regression of the association between COVID-19 vaccination status and residence in the United States compared with other countries.

**(A)**				
**Independent Variable**	**Adjusted Odds Ratio (95% CI)**	**Absolute Vaccinated**	**Absolute Unvaccinated**	***p*-Value**
Country				
● Other countries	1 (Reference)	216 (69.7%)	62 (20.0%)	-
● Chile	1 (Empty)	32 (10.3%)	0 (0%)	-
Age	1.00 (0.97–1.04)	-	-	0.846
Sex				
● Male	1 (Reference)	19 (59.4%)	0 (0%)	-
● Female	0.92 (0.46–1.85)	13 (40.6%)	0 (0%)	0.819
Race				
● Asian	1 (Reference)	0 (0%)	0 (0%)	-
● Black	1 (empty)	0 (0%)	0 (0%)	-
● Hispanic	3.31 (1.03–10.65)	25 (78.1%)	0 (0%)	0.045
● Indigenous	1 (empty)	3 (9.4%)	0 (0%)	-
● Middle Eastern	0.06 (0.001–4.17)	0 (0%)	0 (0%)	0.193
● Other	1 (empty)	0 (0%)	0 (0%)	-
● White	2.01 (0.78–5.19)	4 (12.5%)	0 (0%)	0.149
Education				
● None	1 (empty)	0 (0%)	0 (0%)	-
● Primary complete	0.71 (0.17–3.00)	1 (3.1%)	0 (0%)	0.641
● Some primary	1 (empty)	0 (0%)	0 (0%)	-
● Secondary complete	1 (Reference)	9 (28.1%)	0 (0%)	-
● Some secondary	0.55 (0.20–1.54)	1 (3.1%)	0 (0%)	0.257
● University complete	0.64 (0.27–1.51)	14 (43.8%)	0 (0%)	0.306
● Some university	0.80 (0.27–2.36)	7 (21.9%)	0 (0%)	0.682
Insurance Type				
● Private	11.55 (2.42–55.13)	21 (65.6%)	0 (0%)	0.002
● Public	1 (reference)	11 (34.4%)	0 (0%)	-
● Self-pay	1 (empty)	0 (0%)	0 (0%)	-
COVID Diagnosed				
● No	1 (Reference)	20 (62.5%)	0 (0%)	-
● Yes	7.75 (0.86–70.09)	12 (37.5%)	0 (0%)	0.068
CCI	1.04 (0.75–1.45)	-	-	0.793
**(B)**				
**Independent Variable**	**Adjusted Odds Ratio (95% CI)**	**Absolute Vaccinated**	**Absolute Unvaccinated**	***p*-Value**
Country				
● Other countries	1 (Reference)	212 (68.4%)	18 (5.8%)	-
● China	0.11 (0.03–0.48)	36 (11.6%)	44 (14.2%)	0.003
Age	1.00 (0.97–1.04)	-	-	0.691
Sex				
● Male	1 (Reference)	20 (28.5%)	17 (24.3%)	-
● Female	1.00 (0.49–2.04)	16 (22.9%)	17 (24.3%)	0.999
Race				
● Asian	1 (Reference)	36 (45.0%)	44 (55.0%)	-
● Black	1 (empty)	0 (0%)	0 (0%)	-
● Hispanic	1.02 (0.21–4.94)	0 (0%)	0 (0%)	0.984
● Indigenous	1 (empty)	0 (0%)	0 (0%)	-
● Middle Eastern	0.03 (0.001–1.23)	0 (0%)	0 (0%)	0.064
● Other	1 (empty)	0 (0%)	0 (0%)	-
● White	0.52 (0.12–2.25)	0 (0%)	0 (0%)	0.381
Education				
● None	1 (empty)	0 (0%)	0 (0%)	-
● Primary complete	0.75 (0.17–3.23)	2 (2.5%)	2 (2.5%)	0.699
● Some primary	1 (empty)	0 (0%)	2 (2.5%)	-
● Secondary complete	1 (Reference)	14 (17.5%)	9 (11.3%)	-
● Some secondary	0.72 (0.25–2.09)	9 (11.3%)	8 (10.0%)	0.549
● University complete	0.66 (0.27–1.62)	6 (7.5%)	16 (20.0%)	0.364
● Some university	0.87 (0.29–2.68)	5 (6.2%)	7 (8.7%)	0.815
Insurance Type				
● Private	5.72 (1.10–29.75)	0 (0%)	0 (0%)	0.038
● Public	1 (reference)	36 (45.0%)	44 (55.0%)	-
● Self-pay	1 (empty)	0 (0%)	0 (0%)	-
COVID Diagnosed				
● No	1 (Reference)	36 (45.0%)	44 (55.0%)	-
● Yes	4.82 (0.50–46.80)	0 (0%)	0 (0%)	0.175
CCI	0.86 (0.60–1.23)	-	-	0.404
**(C)**				
**Independent Variable**	**Adjusted Odds Ratio (95% CI)**	**Absolute Vaccinated**	**Absolute Unvaccinated**	***p*-Value**
Country				
● Other countries	1 (Reference)	187 (60.3%)	62 (20.0%)	-
● Singapore	1 (Empty)	61 (19.7%)	0 (0%)	-
Age	1.01 (0.97–1.04)	-	-	0.658
Sex				
● Male	1 (Reference)	37 (60.7%)	0 (0%)	-
● Female	0.97 (0.47–1.99)	24 (39.3%)	0 (0%)	0.929
Race				
● Asian	1 (Reference)	59 (96.7%)	0 (0%)	-
● Black	1 (empty)	0 (0%)	0 (0%)	-
● Hispanic	6.22 (1.95–19.80)	0 (0%)	0 (0%)	0.002
● Indigenous	1 (empty)	0 (0%)	0 (0%)	-
● Middle Eastern	0.12 (0.002–6.10)	0 (0%)	0 (0%)	0.287
● Other	1 (empty)	0 (0%)	0 (0%)	-
● White	3.33 (1.64–8.77)	2 (3.3%)	0 (0%)	0.015
Education				
● None	1 (empty)	1 (1.6%)	0 (0%)	-
● Primary complete	0.87 (0.20–3.81)	1 (1.6%)	0 (0%)	0.853
● Some primary	1 (empty)	0 (0%)	0 (0%)	-
● Secondary complete	1 (Reference)	23 (37.7%)	0 (0%)	-
● Some secondary	0.71 (0.25–2.03)	3 (4.9%)	0 (0%)	0.525
● University complete	0.60 (0.24–1.48)	27 (44.3%)	0 (0%)	0.264
● University some	0.86 (0.28–2.66)	6 (9.8%)	0 (0%)	0.796
Insurance Type				
● Private	11.0 (2.22–54.59)	18 (29.5%)	0 (0%)	0.003
● Public	1 (reference)	18 (29.5%)	0 (0%)	-
● Self-pay	1 (empty)	25 (41.0%)	0 (0%)	-
COVID Diagnosed				
● No	1 (Reference)	41 (67.2%)	0 (0%)	-
● Yes	5.83 (0.61–4.24)	20 (32.8%)	0 (0%)	0.862
CCI	0.90 (0.63–1.28)	-	-	0.554
**(D)**				
**Independent Variable**	**Adjusted Odds Ratio (95% CI)**	**Absolute Vaccinated**	**Absolute Unvaccinated**	***p*-Value**
Country				
● Other countries	1 (Reference)	230 (68.4%)	18 (5.8%)	-
● Switzerland	0.20 (0.05–0.79)	27 (11.6%)	12 (14.2%)	0.022
Age	1.0 (0.97–1.03)	-	-	0.972
Sex				
● Male	1 (Reference)	18 (46.1%)	6 (15.4%)	-
● Female	0.87 (0.43–1.75)	9 (23.1%)	6 (15.4%)	0.69
Race				
● Asian	1 (Reference)	2 (5.1%)	2 (5.1%)	-
● Black	1 (empty)	2 (5.1%)	0 (0%)	-
● Hispanic	4.54 (1.42–14.52)	2 (5.1%)	1 (2.6%)	0.011
● Indigenous	1 (empty)	0 (0%)	0 (0%)	-
● Middle Eastern	0.12 (0.0003–42.63)	0 (0%)	1 (2.6%)	0.479
● Other	1 (empty)	0 (0%)	0 (0%)	-
● White	6.96 (1.69–28.76)	21 (53.9%)	8 (20.5%)	0.007
Education				
● None	1 (empty)	0 (0%)	0 (0%)	-
● Primary complete	0.69 (0.15–3.17)	1 (2.6%)	0 (0%)	0.635
● Some primary	1 (empty)	0 (0%)	0 (0%)	-
● Secondary complete	1 (Reference)	17 (44.8%)	8 (21.1%)	-
● Some secondary	0.51 (0.18–1.40)	4 (10.5%)	2 (5.3%)	0.189
● University complete	0.47 (0.19–1.17)	4 (10.5%)	1 (2.6%)	0.106
● Some university	0.63 (0.20–1.92)	1 (2.6%)	0 (0%)	0.411
Insurance Type				
● Private	11.22 (2.38–52.95)	0 (0%)	0 (0%)	0.002
● Public	1 (reference)	27 (69.2%)	12 (30.8%)	-
● Self-pay	1 (empty)	0 (0%)	0 (0%)	-
COVID Diagnosed				
● No	1 (Reference)	15 (55.6%)	8 (29.6%)	-
● Yes	10.93 (1.14–104.69)	3 (11.1%)	1 (3.7%)	0.038
CCI	1.02 (0.72–1.44)	-	-	0.933
**(E)**				
**Independent Variable**	**Adjusted Odds Ratio (95% CI)**	**Absolute Vaccinated**	**Absolute Unvaccinated**	***p*-Value**
Country				
● Other countries	1 (Reference)	156 (50.3%)	44 (14.3%)	-
● United States	2.24 (0.57–8.80)	92 (29.7%)	6 (1.9%)	0.248
Age	1.0 (0.97–1.03)	-	-	0.995
Sex				
● Male	1 (Reference)	57 (58.2%)	2 (2.0%)	-
● Female	0.90 (0.45–1.80)	35 (35.7%)	4 (4.1%)	0.759
Race				
● Asian	1 (Reference)	17 (17.4%)	1 (1.0%)	-
● Black	1 (empty)	1 (1.0%)	0 (0%)	-
● Hispanic	2.71 (0.71–10.3)	48 (49.0%)	4 (4.1%)	0.144
● Indigenous	1 (empty)	1 (1.0%)	0 (0%)	-
● Middle Eastern	0.05 (0.0003–8.74)	1 (1.0%)	0 (0%)	0.257
● Other	1 (empty)	2 (2.0%)	0 (0%)	-
● White	2.21 (0.86–5.68)	22 (22.4%)	1 (1.0%)	0.101
Education				
● None	1 (empty)	0 (0%)	0 (0%)	-
● Primary complete	0.61 (0.14–2.65)	9 (9.2%)	1 (1.0%)	0.51
● Some primary	1 (empty)	0 (0%)	0 (0%)	-
● Secondary complete	1 (Reference)	22 (22.4%)	1 (1.0%)	-
● Some secondary	0.52 (0.19–1.43)	5 (5.1%)	1 (1.0%)	0.205
● University complete	0.56 (0.23–1.37)	40 (40.9%)	2 (2.0%)	0.205
● Some university	0.74 (0.25–2.22)	16 (16.4%)	1 (1.0%)	0.59
Insurance Type				
● Private	11.07 (2.35–52.27)	46 (46.9%)	2 (2.0%)	0.002
● Public	1 (reference)	42 (42.9%)	4 (4.1%)	-
● Self-pay	1 (empty)	4 (4.1%)	0 (0%)	-
COVID-19 Diagnosed				
● No	1 (Reference)	32 (55.2%)	3 (5.1%)	-
● Yes	8.36 (0.97–72.17)	23 (39.7%)	0 (0%)	0.053
CCI	1.0 (0.72–1.40)	-	-	0.992

CCI: Charlson comorbidity index. Multivariable analyses were adjusted for age, sex, race, education level, insurance type, CCI, and previous COVID-19 diagnosis. (**A**) Adjusted odds ratio comparing vaccination rates between Chile and other countries was not calculated because the rate of vaccination was 100% in Chile. (**B**) Adjusted odds ratio shows that patients in China were significantly less likely to be vaccinated against COVID-19 than patients in other countries. (**C**) Adjusted odds ratio comparing vaccination rates between Singapore and other countries was not calculated because the rate of vaccination was 100% in Singapore. (**D**) Adjusted odds ratio shows that patients in Switzerland were significantly less likely to be vaccinated against COVID-19 than patients in other countries. (**E**) Adjusted odds ratio shows that patients in the US did not have significantly different rates of COVID-19 vaccination compared with patients in other countries. Of note, patients with private insurance were significantly more likely to be vaccinated than those with publicly funded insurance.

### 3.3. Association between Psoriasis Treatment Modality and COVID-19 Vaccination

Compared with all patients on biologics globally, patients on phototherapy had similar vaccination rates (OR: 2.4, 95% CI: 0.18–32.34, *p* = 0.506). However, patients on topical therapy alone were 7.2 times more likely to have received at least one COVID-19 vaccination (OR: 7.2, 95% CI: 1.6–31.6, *p* = 0.009), and patients on oral systemics were 10.9 times more likely to have received at least one COVID-19 vaccination than those on biologics (OR: 10.9, 95% CI: 2.1–56.6, *p* = 0.004). The results of the multivariable analyses for COVID-19 vaccination among psoriasis treatment types are presented in Table 3.

**Table 3:** Multivariable logistic regression of the association between COVID-19 vaccination status and psoriasis treatment regimens compared with biologics.

Independent Variable	Adjusted Odds Ratio (95% CI)	Absolute Vaccinated	Absolute Unvaccinated	*p*-Value
Treatment Type				
● Biologics	1 (Reference)	142 (49.8%)	34 (11.9%)	-
● Oral systemics	10.94 (2.11–56.55)	47 (16.4%)	3 (1.0%)	0.004
● Phototherapy	2.41 (0.18–32.34)	20 (7.0%)	1 (0.3%)	0.506
● Topicals	7.20 (1.64–31.56)	37 (12.9%)	2 (0.7%)	0.009
Age	1.0 (0.96–1.05)	-	-	0.977
Sex				
● Male	1 (Reference)	151 (48.7%)	35 (11.3%)	-
● Female	0.77 (0.32–1.86)	97 (31.3%)	27 (8.7%)	0.556
Race				
● Asian	1 (Reference)	114 (36.8%)	47 (15.2%)	-
● Black	1 (empty)	3 (1.0%)	0 (0%)	-
● Hispanic	0.86 (0.11–7.05)	75 (24.2%)	5 (1.6%)	0.89
● Indigenous	1 (empty)	4 (1.3)	0 (0%)	-
● Middle Eastern	0.14 (0.0006–33.73)	1 (0.3%)	1 (0.3%)	0.484
● Other	1 (empty)	2 (0.6)	0 (0%)	-
● White	3.07 (0.41–23.17)	49 (15.8%)	9 (2.9%)	0.277
Education				
● None	1 (empty)	1 (0.3%)	0 (0%)	-
● Primary complete	1.34 (0.24–7.49)	14 (4.5%)	4 (1.3%)	0.737
● Some primary	1 (empty)	0 (0%)	2 (0.6%)	-
● Secondary complete	1 (Reference)	85 (27.4%)	18 (5.8%)	-
● Some secondary	0.69 (0.19–2.51)	22 (7.1%)	11 (3.5%)	0.575
● University complete	0.74 (0.23–2.41)	91 (29.4%)	19 (6.1%)	0.619
● Some university	0.65 (0.14–3.09)	35 (11.3%)	8 (2.7%)	0.592
Insurance Type				
● Private	1.03 (0.13–8.19)	85 (27.4%)	2 (0.6%)	0.978
● Public	1 (reference)	134 (43.2%)	60 (19.4%)	-
● Self-pay	1 (empty)	29 (9.4%)	0 (0%)	-
COVID-19 Diagnosed				
● No	1 (Reference)	144 (55.8%)	55 (21.3%)	-
● Yes	10.04 (0.70–143.22)	58 (22.5%)	1 (0.4%)	0.089
CCI	0.71 (0.45–1.11)	-	-	0.13
Country				
● Chile	1 (empty)	32 (10.3%)	0 (0%)	-
● China	0.05 (0.004–0.59)	36 (11.6%)	44 (14.2%)	0.018
● Singapore	1 (empty)	61 (19.7%)	0 (0%)	-
● Switzerland	0.04 (0.005–0.34)	27 (8.7%)	12 (3.9%)	0.003
● United States	1 (Reference)	92 (29.7%)	6 (1.9%)	-

CCI: Charlson comorbidity index. Multivariable logistic regression comparing COVID-19 vaccination rate by psoriasis treatment type for adult psoriasis patients. Regression adjusted for country of residence, age, sex, race, education level, insurance type, CCI, and previous COVID-19 diagnosis. Adjusted odds ratio shows that patients with topicals and oral systemics were significantly more likely to be vaccinated against COVID-19 compared with patients on biologics.

## 4. Discussion

Our study revealed in an international comparison that psoriasis patients demonstrate differential vaccination rates against COVID-19 based on country of residence and treatment regimen. These results are among the first to compare the rate of COVID-19 vaccination among patients with psoriasis internationally.

COVID-19 vaccination rates differed between countries. Psoriasis patients from China and Switzerland were significantly less likely to report being vaccinated against COVID-19 compared with those from the other countries included in this study. However, in Switzerland, vaccination rates may have been limited due to data collection taking place early in the pandemic during the height of vaccine approval and distribution. This may explain why the general population of Switzerland and our cohort of psoriasis patients from Switzerland had lower reported vaccination rates compared with other countries.

COVID-19 vaccination rates also differed within each country between the psoriasis patients in our study and the general population of the country. At the time that data collection for our study concluded in October 2022, COVID-19 vaccination rate in our China cohort (45.0%) was substantially lower than that of the general population in China (91.4%) [12]. In Switzerland, COVID-19 vaccination rate in our Switzerland cohort (69.2%) was similar to that of the general population in Switzerland (69.7%) [12]. However, in Chile, Singapore, and the US, the rate of vaccination in our cohort was higher than in the respective general populations: In Chile, 100.0% of our cohort was vaccinated compared with 92.2% of the general population [12]. In Singapore, 100.0% of our cohort was vaccinated compared with 91.2% of the general population [12]. In the US, 93.9% of our cohort was vaccinated compared with 80.2% of the general population [12].

### 4.1. Comparison by Country

These global differences in rates of COVID-19 vaccination may be explained by several factors. In the US, the Federal Drug Administration issued an Emergency Use Authorization for the Pfizer and Moderna vaccines, and guidelines were set by the National Psoriasis Foundation (NPF) to encourage vaccination [2]. In Chile, Singapore, and the US, vaccination was available to each country’s respective citizens for free, and vaccination drives were held at convenient locations [13]. In Singapore and Chile, studies have cited collectivist ideology as a reason for the high vaccination rates in both countries [14,15].

China offered similar strategies for vaccination as described above, yet there was a decreased vaccination rate among psoriasis patients in our cohort compared with the general Chinese public [14]. This difference may be due to regional variations in COVID-19 vaccination in China: Some economically disadvantaged regions have significantly lower vaccination rates compared with more affluent regions [14,16]. It is unknown where respondents in our study population reside within China, but sampling bias may contribute to the discrepancy in our data compared with population-wide reports. More data are needed from a wide range of research facilities to accurately assess the vaccination rate of psoriasis patients within China.

Additionally, Switzerland offered COVID-19 vaccinations for free, but the low vaccination rate in Switzerland compared with other countries in our analyses may be due to lack of availability. COVID-19 vaccines were approved in Switzerland in December of 2020 and administered to priority groups such as essential workers, immunocompromised individuals, and adults older than 80 in the beginning of 2021 [17]. However, there was a lag in distribution due to delegation of financing and implementation of vaccines to the country’s individual cantons rather than a centralized government [17]. We collected a portion of patient data from Switzerland early in the pandemic, prior to widespread availability of COVID-19 vaccines, which may contribute to the low vaccination rates in our cohort.

Finally, patient insurance types varied between countries, but insurance status had minimal impact on patients’ accessibility to COVID-19 vaccines. Switzerland, China, Singapore, and Chile utilized government funds and programs to administer COVID-19 vaccines to patients at no cost [13,18,19,20]. In the US, COVID-19 vaccines were covered by most insurance plans and offered at low or no cost at local immunization programs across the country [21,22].

### 4.2. Comparison by Treatment Group

Patients using topical medication alone or oral systemics were significantly more likely to report at least one COVID-19 vaccination compared with psoriasis patients on biologics. This may suggest that there is some hesitancy among patients on biologics to receive the COVID-19 vaccine.

Patients with immune-mediated inflammatory diseases (i.e., psoriasis) and their providers cite concerns about long-term safety and disease flares as reasons for vaccine hesitancy [23,24,25,26,27]. However, the current literature does not support hypotheses that psoriasis patients on biologics are more likely to experience reduced efficacy of COVID-19 vaccination or that they are at risk for psoriatic flares post-vaccination [28,29,30,31].

The prevalence of treatment groups used by psoriasis patients varied significantly between countries, which may be attributed to differences in psoriasis treatment guidelines, patient preferences and perceptions, and availability and accessibility of certain treatments in each country.

### 4.3. Limitations

A portion of our data was collected early in the COVID-19 pandemic during vaccine distribution, which limited the amount of data and cohort size available in the GHSP. The limited data in certain countries may be a root of bias in our study cohort. Future studies may compare the number of patients who were partially vaccinated, completed a full series, and received a booster vaccine. Additionally, the diversity of our cohort reflects the regions within each country that are participating in our study. As we continue to expand the GHSP, we anticipate larger and more diverse groups of global psoriasis patients in future studies.

Moreover, due to the voluntary nature of survey completion and participation in the study, sampling bias and nonresponse bias played a role in our cohort. Our cohort included patients who were available to take part in our study and willing to answer the questions regarding COVID-19 vaccination status.

## 5. Conclusions

This study is among the first to compare COVID-19 vaccination rates in psoriasis patients globally. Our results indicate a decreased likelihood of COVID-19 vaccination among psoriasis patients living in China and Switzerland compared with patients living in other countries. Additionally, psoriasis patients on biologics are less likely to be vaccinated compared with patients using other treatment modalities. This study highlights the need to increase patient education regarding COVID-19 vaccines. It is important for dermatologists to be aware of this gap in vaccination among psoriasis patients and continually encourage patients to receive COVID-19 vaccines.

## Figures and Tables

**Table 1:** Baseline characteristics of GHSP participants.

Characteristic	Chile	China	Singapore	Switzerland	US	*p*-Value
Age, mean (SEM) years	42.7 (14.6)	41.2 (13.5)	49.2 (15.9)	49.8 (13.9)	52.2 (13.5)	<0.001 *
Male sex, n (%)	18 (58.1)	44 (57.1)	31 (56.4)	24 (61.5)	46 (57.5)	0.999 *
BSA, mean (SEM)	9.3 (14.5)	13.2 (14.7)	4.9 (6.9)	5.5 (7.5)	11.5 (19.2)	0.006 *
DLQI, mean (SEM)	8.2 (5.9)	9.5 (5.9)	5.5 (5.2)	5.3 (5.0)	6.5 (6.9)	0.001 *
PASI, mean (SEM)	7.8 (7.2)	10.2 (7.9)	4.8 (3.3)	4.8 (4.9)	5.0 (7.9)	<0.001 *
PGA, mean (SEM)	1.5 (0.9)	2.4 (0.8)	1.9 (0.7)	1.7 (0.9)	2.0 (1.1)	<0.001 *
Treatment, n (%)						<0.001 †
● Topicals	6 (18.8)	14 (24.6)	8 (13.3)	4 (10.3)	7 (7.1)	
● Phototherapy	5 (15.6)	2 (3.5)	9 (15.0)	1 (2.6)	4 (4.1)	
● Oral systemics	11 (34.4)	12 (21.1)	20 (33.3)	5 (12.8)	2 (2.0)	
● Biologics	10 (31.3)	29 (50.9)	23 (38.3)	29 (74.4)	85 (86.7)	
CCI, mean (SEM)	1.0 (1.2)	0.38 (0.8)	1.72 (1.4)	1.51 (1.6)	1.95 (1.87)	<0.001 *
Vaccinated, n (%)	32 (100.0)	36 (45.0)	61 (100.0)	27 (69.2)	92 (93.9)	<0.001 †
Education, n (%)						<0.001 †
● None	0 (0)	0 (0)	1 (1.6)	0 (0)	0 (0)	
● Primary complete	1 (3.1)	4 (5.0)	1 (1.6)	2 (5.1)	10 (10.2)	
● Primary some	0 (0)	2 (2.5)	0 (0)	0 (0)	0 (0)	
● Secondary complete	9 (28.1)	23 (28.9)	23 (37.7)	25 (64.1)	23 (23.5)	
● Secondary some	1 (3.1)	17 (21.3)	3 (4.9)	6 (15.4)	6 (6.1)	
● University complete	14 (43.8)	22 (27.5)	27 (44.3)	5 (12.8)	42 (42.9)	
● University some	7 (21.9)	12 (15.0)	6 (9.8)	1 (2.6)	17 (17.3)	
Occupation, n (%)						0.095 †
● Homemaker	1 (3.1)	2 (2.5)	0 (0)	2 (5.1)	2 (2.0)	
● Retired	1 (3.1)	10 (12.5)	12 (19.7)	5 (12.8)	16 (16.3)	
● Retired part-time	1 (3.1)	0 (0)	1 (1.6)	0 (0)	0 (0)	
● Student	3 (9.4)	5 (6.3)	2 (3.3)	1 (2.6)	0 (0)	
● Student part-time	1 (3.1)	0 (0)	0 (0)	0 (0)	2 (2.0)	
● Unemployed	2 (6.3)	5 (6.3)	1 (1.6)	3 (7.7)	10 (10.2)	
● Unemployed (health)	0 (0)	0 (0)	0 (0)	0 (0)	3 (3.1)	
● Unemployed (seeking)	0 (0)	1 (1.3)	0 (0)	0 (0)	1 (1.0)	
● Working full time	18 (56.3)	55 (68.8)	38 (62.3)	24 (61.5)	51 (52.0)	
● Working full time (home)	1 (3.1)	0 (0)	2 (3.3)	1 (2.6)	0 (0)	
● Working part time	4 (12.5)	1 (1.3)	5 (8.2)	3 (7.7)	13 (13.3)	
● Working part time (home)	0 (0)	1 (1.3)	0 (0)	0 (0)	0 (0)	
Race, n (%)						<0.001 †
● Asian	0 (0)	80 (100)	59 (96.7)	4 (10.3)	18 (18.4)	
● Black	0 (0)	0 (0)	0 (0)	2 (5.1)	1 (1.0)	
● Hispanic	25 (78.1)	0 (0)	0 (0)	3 (7.7)	52 (53.1)	
● Indigenous	3 (9.4)	0 (0)	0 (0)	0 (0)	1 (1.0)	
● Middle Eastern	0 (0)	0 (0)	0 (0)	1 (2.6)	1 (1.0)	
● Other	0 (0)	0 (0)	0 (0)	0 (0)	2 (2.0)	
● White	4 (12.5)	0 (0)	2 (3.3)	29 (74.4)	23 (23.5)	
Insurance, n (%)						<0.001 †
● Private	21 (65.6)	0 (0)	18 (29.5)	0 (0)	48 (49.0)	
● Public	11 (34.4)	80 (100)	18 (29.5)	39 (100)	46 (46.9)	
● Self-pay	0 (0)	0 (0)	25 (41.0)	0 (0)	4 (4.1)	
Prior COVID-19, n (%)						<0.001 †
● Yes	12 (37.5)	0 (0)	20 (32.8)	4 (10.3)	23 (23.5)	
● No	20 (62.5)	80 (100)	41 (67.2)	23 (58.9)	35 (25.7)	
● Not reported	0 (0)	0 (0)	0 (0)	12 (30.8)	40 (40.8)	

† Chi-square test of the differences among GHSP participants from each of the five listed countries; * One-way ANOVA of the differences among GHSP participants from each of the five listed countries; SEM: standard error of the mean; BSA: body surface area; DLQI: dermatology life quality index; PASI: psoriasis area and severity index; PGA: physician global assessment; CCI: Charlson comorbidity index. Characteristics include both sociodemographic information such as age, race, and highest degree of education as well as clinical characteristics such as psoriasis severity (measured by BSA, DLQI, PASI, PGA scores) psoriasis treatment type, vaccination rate, and prior diagnosis of COVID-19.

## Data Availability

The data presented in this study are available on request from the corresponding author.
